# Supplementary material for: Glutamine supplementation improves the efficacy of miltefosine treatment for visceral leishmaniasis
Source: PLoS Negl Trop Dis. 2020 Mar 26;14(3):e0008125. doi: 10.1371/journal.pntd.0008125 (PMC7138311; doi:10.1371/journal.pntd.0008125)
Supplement: S1 Table — (DOCX) [file pntd.0008125.s001.docx]

| **Supplementary Table S1: List of anti-mouse monoclonal antibodies** | | |
| --- | --- | --- |
| **Antibody** | **Clone** | **Fluorophore** |
| CD11c | N418 | BV605 |
| NK1.1 | PK136 | BV785 |
| CD11b | M1/70 | PE/Cy7 |
| Ly6G | 1A8 | BV711 |
| Ly6C | HK14 | PerCP/Cy5.5 |
| I-A/I-E | M5/114.15.2 | APC |
| CD80 | 16-10A1 | Pacific Blue |
| CD3 | REA641 | PerCP/Cy5.5 |
| CD4 | GK1.5 | APC/Cy7 |
| CD8 | 53-6.7 | BV711 |
| CD19 | 6D5 | BV650 |
| CD62L | MEL14 | BV605 |
| CD44 | IM7 | PerCP/Cy5.5 |
| IL-10 | JES5-16E3 | APC |
| TNF-α | MP6-XT22 | FITC |
| TNF-α | MP6-XT22 | PE/Cy7 |
| IFN-γ | XMG1.2 | FITC |
