# Supplementary material for: Glutamine supplementation improves the efficacy of miltefosine treatment for visceral leishmaniasis
Source: PLoS Negl Trop Dis. 2020 Mar 26;14(3):e0008125. doi: 10.1371/journal.pntd.0008125 (PMC7138311; doi:10.1371/journal.pntd.0008125)
Supplement: S2 Table — (DOCX) [file pntd.0008125.s002.docx]

| **Supplementary Table S2: List of primers used in qRT-PCR** | | |
| --- | --- | --- |
| **Forward** | **Reverse** |  |
| ATCAAGCTGGCTAGGGTAACC | GGCCTTTGACATTTCGGATGA |  |
| TGAACAAAGGCATCAAGCAAATG | CAGTCCAGGGTACGGGTCTT |  |
| ATGTGCGGAATCTTTGCCTAC | AGACCCCTGATAAGGGTTTCG |  |
| AGGAGCTTCGGGACTGTATCC | GGGACATGGTGCATTCCAAAA |  |
| GGGGTGACGAGGTGGAGTA | GTTGGGGTTTGTCCTCTCCC |  |
| AACCATTCACTGAGGTAATCCGA | GGGCTGTTTAGTAGGTTTGGGTA |  |
| GGACCTCCAGATCCCATCCT | GGTTTTCCGTTATCATCCCGGTA |  |
| CCCAACTTCTTCAAGATGGTGG | AGAGGCTCAACACATGGTTGC |  |
| GACAACGTCAGATGGTGTCAT | TGCTTGTGTCAACAAAACAATGT |  |
| TGGCCAGCAAGATTGTGGAGAT | TTTGCGGGTGAAGAGGAAGT |  |
| CTGGATCGAGCTGCTCATC | GTTCACAGCTGTGAGGAGC |  |
