# Supplementary material for: Glutamine supplementation improves the efficacy of miltefosine treatment for visceral leishmaniasis
Source: PLoS Negl Trop Dis. 2020 Mar 26;14(3):e0008125. doi: 10.1371/journal.pntd.0008125 (PMC7138311; doi:10.1371/journal.pntd.0008125)
Supplement: Attachment — Submitted filename: Rebutal_Letter_PNTD_final.docx [file pntd.0008125.s003.docx]

**Response to Reviewers**

Manuscript: PNTD-D-19-01729

**Title: Glutamine supplementation improves the efficacy of miltefosine treatment for visceral leishmaniasis**

Dear Editor(s);

On behalf of all co-authors, I would like to express our gratitude for the comprehensive, constructive and complete revision, which contributed to improve the quality and focus of the manuscript. We also appreciated very much the stimulating words given by the referees along the revision. The changes made on the manuscript were attentive to all those considerations and we feel that the end product is now of much greater scientific value.

**Reviewer #1**

*The study by Ferreira and collaborators provides interesting data indicating that dietary glutamine supplementation may act as a promising adjuvant for the treatment of visceral leishmaniasis. The study is well conducted, but I have some concerns, especially regarding analytical (statistical) procedures. Here are some additional comments.*

- *Is there any reason for choosing C57Bl/6J mice? Wouldn't Balb/c mice have been a more susceptible Leishmania donovani?*

We appreciate the reviewer’s comments and interest in the manuscript. Indeed, both C57Bl/6 and Balb/c mice have been extensively used as experimental models for leishmaniasis. While seminal studies with cutaneous *L. major* have clearly demonstrated a clear distinction between Balb/c as a susceptible and C57Bl/6 as resistant, in visceral disease the latter behave mostly as an intermediately resistant host in contrast to 129/Sv as significantly resistant hosts to infection. We have recently demonstrated that during the first month of infection only minor differences were observed regarding parasite burden and cytokine production in Balb/c and C57Bl/6 mice (Bodhale et al., 2018 Cytokine). Considering the abovementioned and that in our study we focus on the early impact (days 14 and 30 post-infection) of pharmacological inhibition of Gls vs glutamine supplementation in the outcome of leishmania infection, we anticipated that any of these strains would serve the purpose of the study.

- *2) Was the in vitro cytotoxicity assay performed from any previous work? If yes, cite the reference.*

Yes. We have added the following references to the manuscript on the Material and Methods section (Tsagozis et al.,2003 Parasite Immunol and Silvestre et al., 2007 Journal of Immunology)

- *3) Line 166: The route of exposure was intraperitoneal rather than intravenous. What is the reason for this? This detail should be clarified in the manuscript.*

Previous data from our lab have shown positive results regarding *L. donovani* inoculation in mice using intraperitoneal route (Mesquita et al. 2018. Cytokine). Additionally, it was also demonstrated by others that although both inoculation routes (intraperitoneal and intravenous) were able to produce progressive leishmania infection in mice, intraperitoneal inoculation results in a higher homogeneity of infections between animals (Rolão et al. 2004. Acta Tropica). We have added a sentence in the manuscript (line 174-175).

- *4) Line 171: The criteria for choosing the dose administered is unclear.*

Glutamine supplementation is an efficient strategy to maintain glutamine levels during several stress conditions (as infection or exercise) that are associated with depletion of glutamine levels in the body, which can be associated with immunodepression in the affected patients. It is already described that both short and long-term glutamine supplementation is safe for humans (Ziegler et al., 1990 J Parenter Enter Nutr). Oral doses of glutamine (100mg/Kg – 300mg/Kg) is responsible for an acute rise in glutamine levels in the plasma with no documented toxicity. In athletes, the tolerated doses of glutamine without any documented toxicity are higher (258mg/Kg – 570mg/Kg) and acute intakes of glutamine of ∼20–30 g (in 70kg ) seem to be without ill effect in healthy adult humans and no harm was reported in athletes consuming 28 g glutamine every day for 14 days (Gleeson et al., 2008 J Nutr). Doses of up to 0.65 g/kg body mass of glutamine have been reported to be tolerated by patients and did not result in abnormal plasma ammonia levels (Ward et al., 2003 Eur J Clin Nutr). As such, the dose was chosen (500mg/kg) based on the previous reported data in humans and according with the daily intake of glutamine in previous studies with rodents (Mok et al., 2008 Pediatr Res; Badole et al., 2014 Plos One). We have clarified this issue on the Material and Methods section (lines 179-181).

- *5) What is the sample number of animals per group?*

Three independent experiments, n=5 per group.

- *6) Tables 1 and 2 may be reallocated to “supplementary material”.*

*We have reallocated both tables to supplementary material.*

- *7) Statistical analyzes are incorrect. Data should be analyzed using a factorial ANOVA. There are different factors which should be considered (gender, time, treatments). Therefore, the authors must redo the analyzes and present the results appropriately (effect of the factors separately and the interaction between them - present* *F values, degrees of freedom and p values). In addition, ANOVA assumptions must be properly tested.*

The reviewer calls an important point regarding the statistical analysis of the data presented. We partially agree with the reviewer. In each graphic from figures 2B to 5, we only have one dependent variable (either parasite burden, number of cells, % of cytokine, etc.) and one independent variable (treatment). We mentioned that “Both males and females … were used” but among independent experiments. In each independent experiment, each group contained only males or females. Therefore, we have reanalyzed the data using a Kruskal-Wallis non-parametric test followed by a Dunn’s post-test. Also, we have redone the statistic analysis on figures 1B and C, in which we agree with the reviewer, performing a two-way ANOVA followed by a Sidak’s post-test, which primary purpose is to understand if there is an interaction between the two independent variables (infection or not and time) on the dependent variable (transcripts). Finally, figure 1D was analysed using a Mann-Whitney test given that we only intend to compare the two conditions (with and without BPTES treatment) independently of the time of infection. Of note, ANOVA assumptions were analyzed through normality test (Shapiro-Wilk test, p>0,05). The F values, degrees of freedom and *p* values were added in the figure legends. The reanalysis did not alter the major findings of the work, yet we have highlighted in the text the modifications we performed following the new statistical analysis (lines 259-264, 273-278 and 361-364)

**Reviewer #2**

*The manuscript reports on the modulation of glutamine metabolism, in the vertebrate host, by Leishmania donovani and suggests a potential therapeutic use of glutamine in combination with miltefosine for the treatment of visceral leishmaniasis. The modulation of the metabolism of the host cells by these parasites is a relevant issue for the understanding of the immune mechanisms that control the infection. However, this topic is little studied. In this sense, the manuscript addresses an interesting topic and provides important new insights into the crosstalk between glutamine metabolism and T cell-mediated immune response to L. donovani infection. Overall, the manuscript is very well written and the data are well presented. Conclusion could be more cautious as suggested below.*

- ***METHODS:*** *The objectives of the study are clearly articulated with the hypothesis stated, the study is well designed and the ethical requirements are being met. As suggested in the major comments for the authors, the methodology for transcriptome analysis must be better detailed. On this regard, information is missing.*

- ***CONCLUSIONS:*** *The conclusions are supported by the data presented. However, it is suggested to moderate some of them in order to avoid reductionisms. Limitations of analysis were not described.*

As mentioned below, we have added the methodology for transcriptomic anaçysis and moderated the discussion.

Major comments:

*Lines 181-187. Detailed information on the transcriptomic analysis is lacking. Information about whether it was a time course analysis, BM or peritoneal macrophages? Number of experiments? RNA Information, such as it was total or poly-A RNA, if it was treated for depletion of ribosomal DNA, how it was isolated and quantified, which quality control was used. How library was constructed? This information must be included for the correct interpretation of the data by the reader. In addition, whether transcriptomic assays were done at 6, 24 and 48 hours or if they were done just at 6h post-infection and the other time points refer to other assays of gene expression analysis. The design of transcriptomic analysis must be detailed.*

We appreciate the reviewer’s comments and interest in the manuscript. We have added the requested information (lines 192-213).

- *Lines 286-287. It would be probably because those cells stopped proliferating due to the lack of glutamine? Please elaborate on that. Lines 297-298. How do authors explain that there was no difference between the untreated and the group supplemented with glutamine? Shouldn't there be an improvement in animals that received supplementation compared to untreated ones?*

The hypothesis raised by the reviewer is probably correct. Previous papers have demonstrated that the depletion of glutamine blocks T cell proliferation and cytokine production (Carr EL, J Immunol, 2010; Johnson MO, Cell, 2018). In our co-culture system, it is plausible that a decreased bioavailability of glutamine is significantly hampering the cytotoxicity. In line with this reasoning, we demonstrate that reduced numbers of CD4 and CD8 T cells in the spleen of infected mice were observed by inhibiting glutamine metabolism *in vivo* through the administration of BPTES. Furthermore, the impact of glutamine in T cell proliferation and cytokine production can also be an explanation of improved cytotoxicity when glutamine supplementation is provided in the *in vitro* system. We have elaborated on this on the new manuscript (lines 402-405).

Regarding the impact of glutamine supplementation, we demonstrate that glutamine supplementation only provides an adjuvant effect once in synergy with miltefosine. Indeed, glutamine supplementation *per se* do not improve the diseases in infected animals. Therefore by maintaining at lower levels parasite burden due to the direct effect of parasite killing-mediated by miltefosine, glutamine may reinforce the immune system, or because it has been proposed that miltefosine is modulating the host immunity by activating Th1 cytokines particularly represented by increased gamma interferon (IFN-γ) and interleukin 12 (IL-12) (Palic S, 2019 Antimicrob Agents Chemother), glutamine synergy may act in synergy. Given that T cell activation is absolutely dependent on extracellular glutamine (Carr EL, J Immunol, 2010), the bioavailability of glutamine will be crucial for the fitness of T cell response and thus, in this case, glutamine supplementation will be relevant. We have elaborated on this on the new manuscript (lines 444-453).

- *Lines 318-320 and figure 3. The supplemented group also decreased the number of CD4 and CD8 T cells. Authors expected that? How to explain this? Shouldn't it be at least the same as the untreated? Or even increase thanks to supplementation? Also, how to explain the reduction of IFN-γ- and TNF produced by CD4 T cells? The authors are encouraged to elaborate on this result.*

Although a tendency was observed particularly in CD4 T cells, no statistical difference was observed in any of the parameters evaluated for both T cells comparing the untreated versus glutamine supplementation groups. Moreover, a tendency, although not significant, of increased number of CD8 T cells producing IFN and TNF is also observed. As mentioned above, glutamine supplementation is only effective as adjuvant in the presence of chemotherapy, and as such, the absence of differences between untreated and glutamine supplemented groups is not surprising.

- *For this reviewer, the conclusions need to be more moderate because, as it is written, it seems to reduce the complexity the parasite-host relationship. It would be ideal if supplementation with glutamine was sufficient to contribute for controlling the infection, but that would be reductionist, since many other factors contribute to the control of the parasite or, conversely, to the establishment of the infection. In fact, parasites also consume glutamine to maintain their proliferation, and a medium supplemented in this amino acid could also result in parasite survival and establishment within the cells. This aspect deserves to be mentioned by the authors. Could parasites compete with macrophages for glutamine? Please elaborate on that and moderate conclusions.*

The reviewer calls an important point regarding the complexity of host-pathogen relationship. Indeed, parasite itself can consume glutamine to maintain proliferation. Yet, our data demonstrate that glutamine-miltefosine synergy significantly reduces the parasite burden while improving the immune response, particularly the CD8 effector response. We have addressed the raised issues and moderated our conclusions on the manuscript (430-453).

Minor points:

- *Figure 3. Captions of this figure should be standardized, i.e., use the same font size.*

We have homogenised the font size on the figures.
